# Poorer and more densely populated regions have lower vaccination capability against COVID-19

**DOI:** 10.17179/excli2022-4798

**Published:** 2022-03-21

**Authors:** Paulo Ricardo Martins-Filho, Ricardo Ruan Rocha Santana, Victor Santana Santos, Lorena G. Barberia

**Affiliations:** 1 Investigative Pathology Laboratory, Health Sciences Graduate Program, Federal University of Sergipe, Aracaju, Sergipe, Brazil; 2 Department of Medicine, Federal University of Sergipe, Lagarto, Sergipe, Brazil; 3 Department of Political Science, University of Sao Paulo, Sao Paulo, Brazil

## ⁯

Studies have discussed the need to improve the production and delivery capacity of vaccines against COVID-19 in poor countries (Maxmen, 2021[5]; Mahmud-Al-Rafat et al., 2022[1]). Unfortunately, COVID-19 has disproportionately affected the most vulnerable regions worldwide, including the opportunity for vaccination. Brazil has been one of the countries with the highest incidence and mortality rates from COVID-19. Although the country has a long history of successful immunization programs, the vaccination campaign against COVID-19 has experienced several setbacks, including politically motivated delays in vaccine procurement, limited investments in local production capabilities, and the federal authorities' unsupported allegations that vaccines are ineffective and unsafe (Martins-Filho and Barberia, 2022[3]). 

Problems that hinder vaccination are heterogeneous among low- and middle-income countries and range from political opposition to resource limitations and the organization of the health systems. In Brazil, the decentralization of the health system requires municipalities to be responsible for the delivery of most primary care services, including vaccination. Therefore, low-income municipalities with fewer health resources may have a lower vaccination capability of their population especially during a pandemic. Vaccination capability can be measured from the doses applied to the population by the number of doses available in given period.

In this letter, we showed the case of Sergipe state, Northeast Brazil, a region with the largest concentration of highly vulnerable people in the country. Sergipe is the smallest Brazilian state with 21,910 km^2^, has an estimated population of ~2.3 million inhabitants, a population density of 105.8 inhabitants/km^2^, and a Human Development Index (HDI) of 0.665. The state is divided into 75 municipalities with an average population density ranging from 17.4 to 3140.6 inhabitants/km^2^. Municipalities can be categorized into three population density strata (low: <50 inhabitants/km^2^; moderate: 50-99 inhabitants/km^2^; and high: ≥100 inhabitants/km^2^) and two levels of HDI (moderate to high HDI-M: 0.600 - 0.799 [33 municipalities]; and low HDI-M: 0.500 - 0.599 [42 municipalities]). 

In Sergipe state, the COVID-19 vaccination campaign started on January 19, 2021. Until 25 February 2022, 1,880,501 vaccine doses for complete vaccination were made available and 1,654,333 doses were applied. The vaccination capability was 88 % (ranging from 74.8 % to 100 %) and vaccination coverage was 71.3 % (ranging from 58.1 % to 81.3 %). Using a backward regression linear model considering the assumptions of normality and homoscedasticity, and a 5 % significance level, we found a lower vaccination capability in municipalities with lower HDI-M (β = 60.1, p = 0.021) and higher population density (natural log-transformed) (β = -2.7, p = 0.014) (Supplementary Figure 1). 

Previously, we had already demonstrated that poorer and more densely populated regions have higher incidence and mortality rates from COVID-19 (Martins-Filho et al., 2020[4]; Martins-Filho, 2021[2]). The present data indicate that these regions also have a lower capability to speedily deploy health resources, specifically in relation to mass vaccination of the population. Lower vaccination capability can lead to wasted doses and lower vaccine coverage, increasing unnecessary expenditures and allowing more people to remain susceptible to the virus.

## Declaration

### Authors' contributions

All authors contributed equally to this work. 

### Conflict of interest statement

The authors have no conflicts of interest to declare.

### Role of funding source

There is no funding source. 

## Supplementary Material

Supplementary information

## References

[1] Mahmud-Al-Rafat A, Hewins B, Mannan A, Kelvin DJ, Billah MM (2022). COVID-19 vaccine inequity, dependency, and production capability in low-income and middle-income countries: the case of Bangladesh. Lancet Infect Dis.

[2] Martins-Filho PR (2021). Relationship between population density and COVID-19 incidence and mortality estimates: A county-level analysis. J Infect Public Health.

[3] Martins-Filho PR, Barberia LG (2022). The unjustified and politicized battle against vaccination of children and adolescents in Brazil. Lancet Reg Health Am.

[4] Martins-Filho PR, de Souza Araújo AA, Quintans-Júnior LJ, Santos VS (2020). COVID-19 fatality rates related to social inequality in Northeast Brazil: a neighbourhood-level analysis. J Travel Med.

[5] Maxmen A (2021). The fight to manufacture COVID vaccines in lower-income countries. Nature.

