# Supplementary material for: Poorer and more densely populated regions have lower vaccination capability against COVID-19
Source: EXCLI J. 2022 Mar 21;21:621–2. doi: 10.17179/excli2022-4798 (PMC9203990; doi:10.17179/excli2022-4798)
Supplement: Supplementary information [file EXCLI-21-621-s-001.pdf]

EXCLI Journal 2022;21:621-622 – ISSN 1611-2156
Received: February 26, 2022, accepted: March 16, 2022, published: March 21, 2022

Supplementary information to:

Letter to the editor:

POORER AND MORE DENSELY POPULATED REGIONS HAVE LOWER VACCINATION CAPABILITY AGAINST COVID-19

Paulo Ricardo Martins-Filho[1], Ricardo Ruan Rocha Santana[2], Victor Santana Santos[2], Lorena G. Barberia[3]

[1] Investigative Pathology Laboratory, Health Sciences Graduate Program, Federal University of Sergipe, Aracaju, Sergipe, Brazil
[2] Department of Medicine, Federal University of Sergipe, Lagarto, Sergipe, Brazil
[3] Department of Political Science, University of Sao Paulo, Sao Paulo, Brazil

* **Corresponding author:** Prof. Paulo Ricardo Martins-Filho, Universidade Federal de Sergipe, Hospital Universitário. Rua Cláudio Batista, s/n. Bairro Sanatório. Aracaju, Sergipe, Brasil. CEP: 49060-100. E-mail: prmartinsfh@gmail.com

https://dx.doi.org/10.17179/excli2022-4798

This is an Open Access article distributed under the terms of the Creative Commons Attribution License (http://creativecommons.org/licenses/by/4.0/).

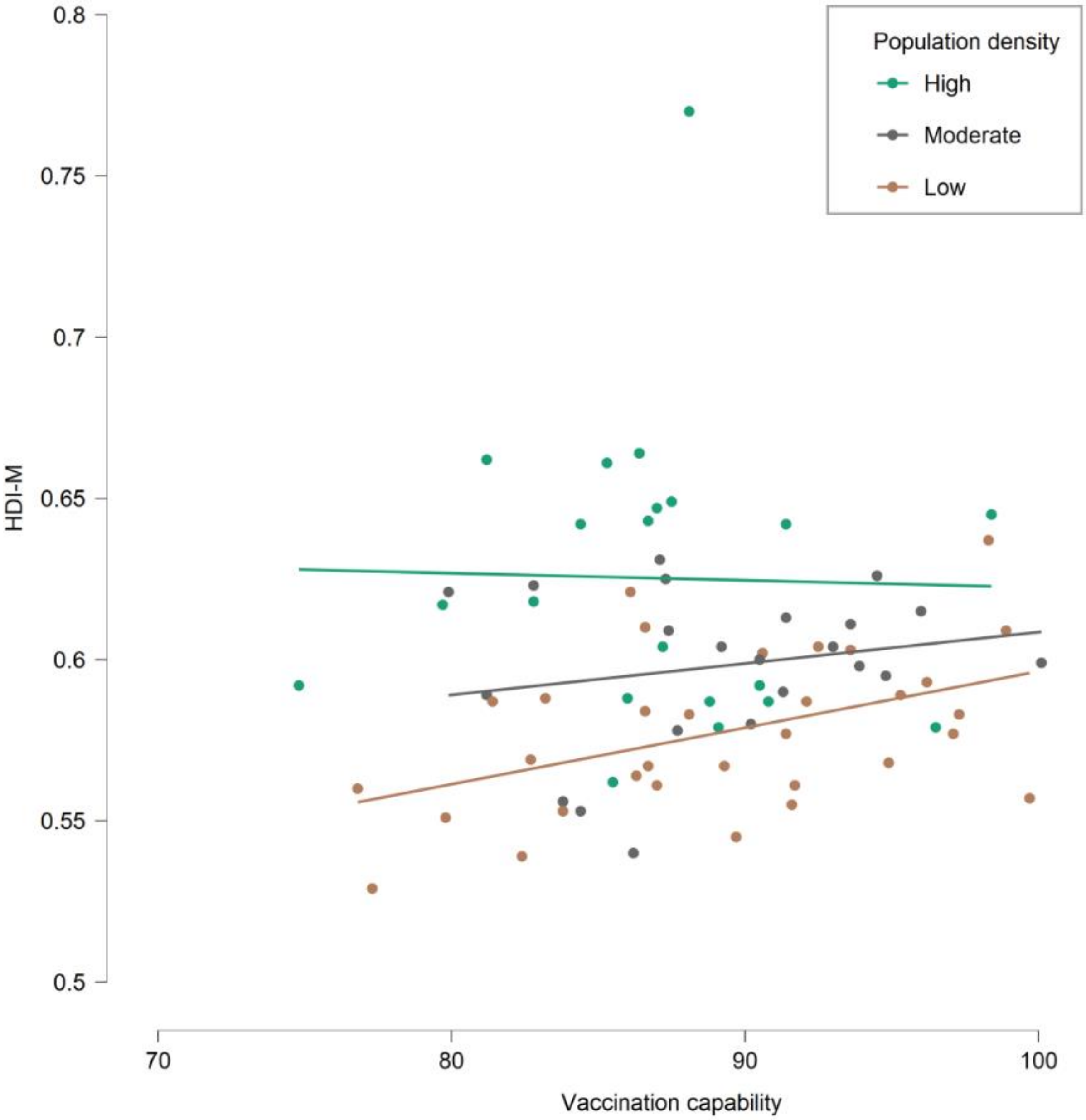

Supplementary Figure 1: Vaccination capability according to HDI-M and population density in Sergipe state, Northeast Brazil
